# Supplementary material for: Rapid turnover of life-cycle-related genes in the brown algae
Source: Genome Biol. 2019 Feb 14;20:35. doi: 10.1186/s13059-019-1630-6 (PMC6374913; doi:10.1186/s13059-019-1630-6)
Supplement: Supplementary file 3 — Protein alignments, corresponding Gblocks html file and CDS sequences for the lineage conserved gametophyte-biased genes. (ZIP 2109 kb) [file 13059_2019_1630_MOESM3_ESM.zip › Con_GA_alignments/Ec-14_004660.aa_ali.fasta-gb.htm]

Ec-14\_004660.aa.fasta.trans.aa\_ali.fasta


## Gblocks 0.91b Results

Processed file: **Ec-14\_004660.aa.fasta.trans.aa\_ali.fasta**  
Number of sequences: **2**  
Alignment assumed to be: **Protein**  
New number of positions: **224** (selected positions are underlined in blue)

```
                         10        20        30        40        50        60
                 =========+=========+=========+=========+=========+=========+
Ec-14_004660     MRPGSRGYVGGGLQPPGTGMRMATGQRGGRPMSGRMRTGQVTAGPSREAAYGVALSQEVK
MSTRG.14838.1    ----------------LAAMKLQS---------------------------EVAVRQQE-
                                                                             


                         70        80        90       100       110       120
                 =========+=========+=========+=========+=========+=========+
Ec-14_004660     VTDRPVTQQGMSGMKTAGGGGGGGGGGGRQVQDGSFFIGVLRSKMNDVRAELGRLQAEID
MSTRG.14838.1    -TDRLSSQISHLEARAAAQEGGGGGGGS--------------------------------
                  ##########################                                 


                        130       140       150       160       170       180
                 =========+=========+=========+=========+=========+=========+
Ec-14_004660     RHEKSVSQQGHLERTYDTMLQDVKALEGTLADYNLAVDKARTTADPGEVTKYLHDLEDRN
MSTRG.14838.1    ------------------------------------------------------------
                                                                             


                        190       200       210       220       230       240
                 =========+=========+=========+=========+=========+=========+
Ec-14_004660     RREAQGLDEIFLAKQQHERETQELEAEMEAVHGRMEEKINTLEPEKLHEYRVLLQRNNHL
MSTRG.14838.1    ------------------------------------------------------------
                                                                             


                        250       260       270       280       290       300
                 =========+=========+=========+=========+=========+=========+
Ec-14_004660     QAEVAAKQAEADRLSAMIGQHESRSQGAGGAALRGEYQVLEKSVGALRRERENLSQDLEI
MSTRG.14838.1    ---------------------------GGAAALRAAYNQLEKSVGGLRRERENLQQDLEI
                                             ################################


                        310       320       330       340       350       360
                 =========+=========+=========+=========+=========+=========+
Ec-14_004660     SNMNPKDARDRLLSKVKADQERLKQLDARAAETTEEVVRMKERLEDLESDLKADRKG---
MSTRG.14838.1    SNMNPKEARERLLSKVKADQERLKQLEQRTVETSGVVLGMKERLQDLESDLKADRKGGGG
                 #########################################################   


                        370       380       390       400       410       420
                 =========+=========+=========+=========+=========+=========+
Ec-14_004660     DVAQNKVEVLQKRDKEMTDFIEKFDQTKQEALDDQRSARSTIVGLLEHISTGLESQHHIP
MSTRG.14838.1    DVSQNKLEVLQKRDKEMTDFMDKFNQTREEALSDQTSARSTIVAVLEHISTGLESQHHIP
                 ############################################################


                        430       440       450       460       470       480
                 =========+=========+=========+=========+=========+=========+
Ec-14_004660     DKGGMREMEDEKSFKDKQLKSSEATMRRLHQDKEKRLQEMEKVKTLDEKIPLELNNLKEK
MSTRG.14838.1    DKGKLKEMEDEQSFKDKQLKSSEATMRRLHQDKEKRLQEMEKVNTLDEKV----------
                 #################################################           


                        490       500       510       520       530       540
                 =========+=========+=========+=========+=========+=========+
Ec-14_004660     MDRMRREMREFDDIDGLRDEAAQTKEHLQGLRKQYRWRVEAMRGQVQQLTARHEACKKEL
MSTRG.14838.1    ---------------------------L--------------YGG---------------
                                                                             


                        550       560       570       580       590       600
                 =========+=========+=========+=========+=========+=========+
Ec-14_004660     AASETGKTLEALERKMRTYETNIFFLKEFVETKGREIDFQSLRGECMGMVKDLNEAVKRK
MSTRG.14838.1    VASR--------------------------------------------------------
                                                                             


                        610
                 =========+
Ec-14_004660     GETEGFGVAY
MSTRG.14838.1    -----IGNKP
```

```
Parameters used
Minimum Number Of Sequences For A Conserved Position: 2
Minimum Number Of Sequences For A Flanking Position: 2
Maximum Number Of Contiguous Nonconserved Positions: 8
Minimum Length Of A Block: 10
Allowed Gap Positions: None
Use Similarity Matrices: Yes
```

```
Flank positions of the 3 selected block(s)
Flanks: [62  87]  [269  357]  [361  469]  

New number of positions in Ec-14_004660.aa.fasta.trans.aa_ali.fasta-gb:  224  (36% of the original 610 positions)
```
